# Prevalence of dens invaginatus in young Israeli population and its association with clinical morphological features of maxillary incisors

**DOI:** 10.1038/s41598-020-74396-z

**Published:** 2020-10-13

**Authors:** Anda Kfir, Nurit Flaisher Salem, Lobna Natour, Zvi Metzger, Noa Sadan, Shlomo Elbahary

**Affiliations:** 1 Department of Endodontology, The Goldschleger School of Dental Medicine, Tel Aviv University, Ramat Aviv, Tel Aviv, Israel; 2 Department of Orthodontics, The Goldschleger School of Dental Medicine, Tel Aviv University, Tel Aviv, Israel

**Keywords:** Anatomy, Oral anatomy

## Abstract

Dens invaginatus is an anomaly mostly observed in maxillary incisors. This study aimed to assess the prevalence of dens invaginatus in maxillary incisors in young Israeli population and to study its potential association with clinical coronal morphological features. Data was collected from periapical radiographs and clinical photographs of patients from Orthodontics Department between 2006 and 2018. Radiographic characteristics were evaluated and compared to clinical coronal morphological features. Statistical analysis was performed using the Pearson chi-square test with statistical significance set at *p* < 0.05. The sample included 1621 maxillary incisors from 547 patients. Dens invaginatus was observed in 422 (26%) of these teeth. Maxillary lateral incisors were more affected than central incisors. In 103 patients dens invaginatus was unilateral, while in all other cases it was bilateral. Unique clinical morphological characteristics were observed in 88% of the teeth that exhibited radiographic evidence of dens invaginatus. Dens invaginatus Type I was most frequently observed, accounting for 90% of the teeth. A significant association between clinical coronal morphological features and dens invaginatus was detected. Dens invaginatus is common in maxillary incisors of the study population. Several clinical morphological features may predict the presence of dens invaginatus.

## Introduction

Dens invaginatus is a structural anomaly that may be found in any tooth but is mainly observed in anterior maxillary teeth^1–6^. Its severity may range from a very small change in tooth structure to dramatic and complicated abnormalities in tooth structure^4,7^. The etiology of invaginated teeth is not clear. A variety of forces that may have been applied to the tooth germ during development, which may apply pressure on the enamel organ, have been suggested as causal elements for dens invaginatus^8^, These include: adjacent tooth germs, trauma, infection, focal growth retardation of the tooth bud, focal growth acceleration of the tooth bud, and restriction of the dental arch. Oehlers^9^ introduced a classification based on the radiographic appearance of the invagination and its severity: Type I dens invaginatus is an enamel-lined minor form of invagination that occurs within the confines of the crown, without extending beyond the cemento-enamel junction; Type II dens invaginatus is an enamel-lined invagination that invades the root but remains confined as a blind sac, with or without communication with the dental pulp; and Type III is an invagination that penetrates through the root with an opening at the apical area and a ‘second foramen’ in the apical or periodontal area, without immediate communication with the pulp. The invaginations may be completely lined by enamel, but frequently, cementum lines the invagination. This classification is commonly used by researchers in this field and was also used in the present study^10^.

An invaginated tooth may exhibit no clinical signs of anomaly; nevertheless, there are several clinical morphological features that have previously been randomly reported to appear together with dens invaginatus. Tooth may appear normal, aside from an abnormally deep lingual pit accompanied by a slight overdevelopment of the cervico-lingual ridge^9^. The crown may occasionally be conical, such as a caniniform or peg-shaped tooth with an incisal pit leading to an invagination^9^. In some cases, the labio-lingual or mesio-distal diameter of the crown may be increased in such teeth^10,11^. Lingually, there may be an exaggerated cingulum that is often referred to as "talon cusp" or lingual "tubercle"^9^. A bifid cingulum may also be associated with the presence of dens invaginatus^10^. In rare cases, dens invaginatus may be present together with incisal notching in association with a labial groove^11^. The association of these clinical morphological features with radiological presence of dens invaginatus was evaluated in the present study. Such variations of tooth morphology is of special importance to the Pediatric dentistry because when dens invaginatus is detected early, simple preventive measures may be applied to avoid further complications, which may range from dental caries in the invagination, all the way to involvement of the pulp, and to complicated and extremely challenging endodontic cases^7^.

The gold standard for the detection of dens invaginatus is radiography. However, screening young patients who do not present any apparent pathology, such as caries or fractures, by taking radiographs of maxillary anterior teeth is not considered an acceptable practice. On the other hand, if the abovementioned coronal morphological features are shown to be associated with dens invaginatus in a large-scale study, such morphological features may serve as an important tool for the selection of patients who may benefit from diagnostic evaluation with periapical radiography, thereby allowing for the early detection of dens invaginatus.

The one field of pediatric dentistry in which taking radiographs of the maxillary anterior teeth is mandatory and common practice is orthodontics. Therefore, patient data was acquired from the Orthodontic Clinic at Tel Aviv University for the present study. High-quality photographs of the anterior teeth are also routinely taken at the abovementioned clinic. This allowed for a study of both the prevalence of dens invaginatus and its potential association with clinical coronal morphological features. Of note, the present study was conducted without any unjustified exposure of the young study population to additional radiation.

The present study was conducted to assess the prevalence of dens invaginatus in maxillary incisors of a young Israeli population and to study the potential associations between dens invaginatus and clinical morphological features of these teeth.

## Materials and methods

The study material was composed of all orthodontic records of young Israeli patients admitted to the Department of Orthodontics at Tel Aviv University between 2006 and 2018 The ethics committee of the university approved the study (Approval 35.18).

The inclusion criteria were dental records with both good periapical radiographs taken from the ortho-radial direction without overlap with adjacent teeth and good clinical digital photographs of the maxillary incisors in which the lingual side of the tooth could clearly be seen. Furthermore, there had to be at least two comparable periapical radiographs for both sides of the jaw.

Subjects with craniofacial developmental anomalies, such as a cleft lip, were excluded from this study because of the established association of these syndromes with missing teeth, impacted teeth, and teeth exhibiting delayed eruption. Patients with traumatized, carious, restored, or fractured teeth and patients without satisfactory periapical radiographs or satisfactory clinical images were also excluded from the study.

Initially, 616 patient records were considered for inclusion into the present study. However, 69 records were excluded from the study due to the abovementioned exclusion criteria; therefore, 547 records were included in the present study. These records included a total of 1621 maxillary incisors that answered to all the above inclusion criteria and were examined for the presence of dens invaginatus and clinical anatomical features of the crown.

### Radiographic presentation

Each radiograph was randomly presented for evaluation in a semi-dark room using a high-resolution LCD monitor. They were independently examined by two examiners at different times, and a combined decision was later made on whether the teeth had dens invaginatus.

**Figure 1:**
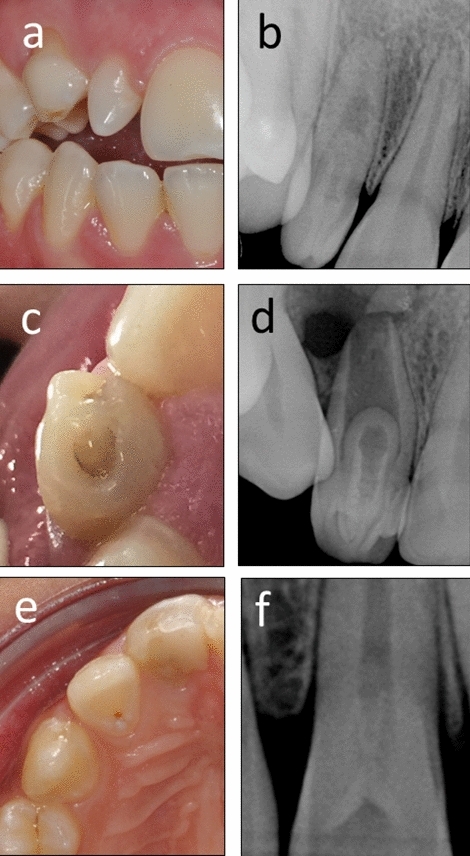
Coronal anatomical features associated with dens invaginatus. (**a**,**b**) Conical, peg-shaped maxillary lateral incisor with a Type II dens invaginatus. (**c**,**d**) Bifid cingulum of a maxillary central incisor with a Type II dens invaginatus. (**e**,**f**) Maxillary lateral incisor with a deep lingual pit and Type I dens invaginatus. No Type III dens invaginatus cases were found in the present study.

The invaginations observed on the periapical radiographs were recorded and categorized by the Oehlers classification system^4^, which is based on the extent of the invaginated dental tissue as follows: Type I: the invagination was confined within the crown, extending only to the cemento-enamel junction (Fig. 1f); Type II: the invagination extended apically beyond the cemento-enamel junction (Fig. 1b,c); and Type III: the invagination extended beyond the cemento-enamel junction and exhibited a second “foramen” into the peri-apical tissue or the lateral periodontal ligament^4^ (Fig. 3c).

Prior to the present investigation, both examiners were calibrated. Each examiner read 100 radiographs containing 10 different cases of dens invaginatus. Two weeks after this initial training, the examiners reread a sample of 12 radiographs containing cases of dens invaginatus, and there was a 100% agreement between the examiners regarding the detection and classification of dens invaginatus. Thus, complete inter- and intra-examiner reliability was established.

The prevalence and distribution of radiographically proven dens invaginatus were calculated with respect to the tooth type, age and gender of the patients.

### Morphological presentation

The clinical features of teeth with dens invaginatus were evaluated from clinical digital photographs. Anatomical features were recorded and categorized based on the following criteria:

- Normal crown appearance.
- Increased labio-lingual or mesio-distal diameter of the crown.
- Conical crown, such as a caniniform or peg-shaped tooth with an incisal pit leading to an invagination (Fig. 1a).
- Exaggerated cingulum, which is often referred to as a "talon cusp" or lingual "tubercle".
- Bifid cingulum (Fig. 1c).
- Incisal notching with a labial groove.
- An almost normal tooth appearance aside from an abnormally deep lingual pit (Fig. 1e) accompanied by a slight overdevelopment of the cervico-lingual ridge.

### Statistical analysis

For statistical analysis, the clinical features were grouped as follows (Table 1): Group a: Normal crown appearance; Group b: Altered crown shape and dimensions—the labio-lingual or mesio-distal diameter of the crown is increased, or the crown is conical, such as a canine-like or peg-shaped tooth; Group c: Developmental grooves, pits and fissures—the tooth appearance is almost normal aside from an abnormally deep lingual pit, accompanied by a slight overdevelopment of a cervico-lingual ridge or incisal notching; Group d: Exaggerated cingulum—an exaggerated cingulum is present; Group e: Bifid cingulum.

**Table 1:** Association of clinical morphological features with dens invaginatus in maxillary incisors.

Crown morphology	Type l DI	Type ll DI	Total teeth with DI with a given crown morphology
a-Normal	43 (84.3%)^†^	8 (15.7%)^†^	51 (3.2%)^§^
b-Size and shape	30 (96.8%)	1 (3.2%)	31 (2.0%)
c-Pits and fissures	179 (89.9%)	20 (10.1%)	199 (12.2%)
d-Large cingulum	30 (81.1%)	7 (18.9%)	37 (2.3%)
e-Bifid cingulum	98 (94.2%)	6 (5.8%)	104 (6.4%)
Total	380 (23.1%)^#^	42 (2.9%)^#^	422 (26.0%)^#^

*DI* Dens invaginatus, identified in periapical radiographs.

^†^Number of teeth with a given type of dens invaginatus that presented a given morphology, and the percent of teeth with that type of DI that presented a given morphology.

^§^Total number of teeth with dens invaginatus that presented a given morphology, and the percent from the total sample of 1621 maxillary incisors studied.

^#^Total number of teeth with a given type of dens invaginatus, presented as percent from the total sample of 1621 maxillary incisors studied.

The statistical analysis included descriptive statistics and frequencies. Fisher’s exact test was used to evaluate the association of dens invaginatus with gender, and the McNemar’s test was used to compare the prevalence of dens invaginatus in the lateral versus central incisors, and the association of the left or right sides with the presence of dens invaginatus. Crosstabs with Pearson chi-square analysis was used to evaluate the association of coronal morphological features with the presence of dens invaginatus. Statistical significance was set as *p* < 0.05.

### Ethical approval

All procedures performed in studies involving human participants were in accordance with the ethical standards of the institutional and/or national research committee and with the 1964 Helsinki declaration and its later amendments or comparable ethical standards.

### Informed consent

Informed consent was obtained from all individual participants included in the study, as part of their acceptance to the Orthodontic Clinic.

## Results

At least one invaginated tooth was present in 37% (n = 204/547 patients) of the sample records.

Although the male/female ratio in the sample was 1:1.29, the rate of dens invaginatus was similar among males (47.99% of teeth) and females (52.01% of teeth). No association was found between dens invaginatus and gender.

A total of 422 teeth (26%, n = 422/1621 teeth) with dens invaginatus were identified. Maxillary lateral incisors were the most commonly affected teeth (61%, n = 258/422 teeth) followed by maxillary central incisors (39%, n = 164/422 teeth) (*p* < 0.05).

Among 204 patients who presented dens invaginatus 103 had a unilateral invagination (50.5%), while the rest had bilateral invaginations.

No associations were identified between invaginated teeth on the left and right sides of the maxilla. Most of the invaginated teeth were associated with one of the clinical morphological characteristics (88.0%, Table 1). Pearson chi-square test showed a significant association between the presence of one of the morphological clinical features and the radiographic presence of dens invaginatus (*p* < 0.05).

Among the affected teeth, Type I dens invaginatus was found in 90% of the affected teeth (n = 380/422), and the rest of the teeth exhibited Type II dens invaginatus (10%, n = 42/422) (Fig. 1b,c). None of the patients’ evaluated teeth in this study presented Type III dens invaginatus.

## Discussion

Our findings indicate that dens invaginatus is a relatively common anomaly in the maxillary incisors of the young Israeli population. Furthermore, significant association between clinical morphological features and the presence of dens invaginatus could clearly be seen. This association suggests that periapical radiographs should be taken of teeth with abnormal morphologies to establish whether dens invaginatus is present; if so, early measures could be taken to prevent future complications. It is likely that the pediatric dentist will be the first to recognize these abnormal morphologies, verify radiographically whether the tooth does have a dens invaginatus and apply the necessary preventive measures, as required.

**Figure 2:**
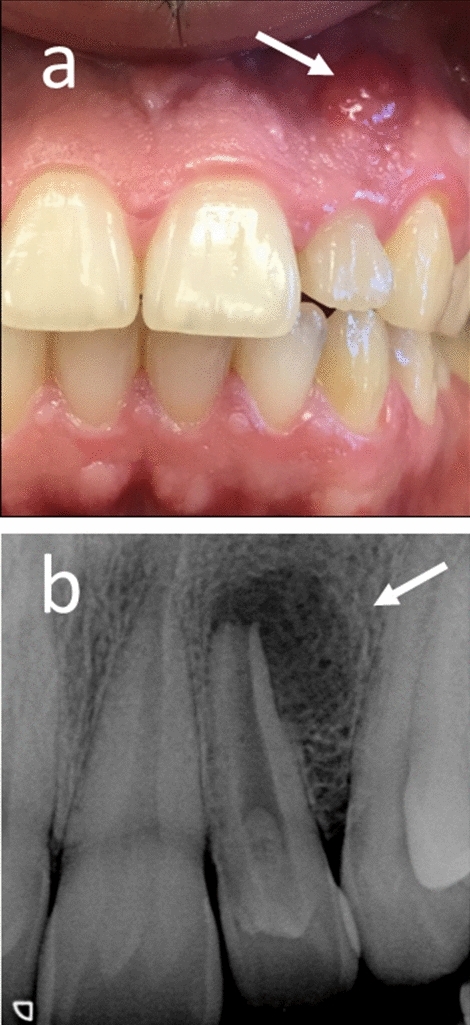
Complication: failure to diagnose dens invaginatus in time. Failure to diagnose in time the dens invaginatus and failure to apply early preventive measures led to pulp involvement, infection and necrosis, resulting in formation a periapical lesion. (**a**) White arrow indicates a suppurating sinus tract (**b**) White arrow: periapical bone resorption in response to infection in the root canal.

**Figure 3:**
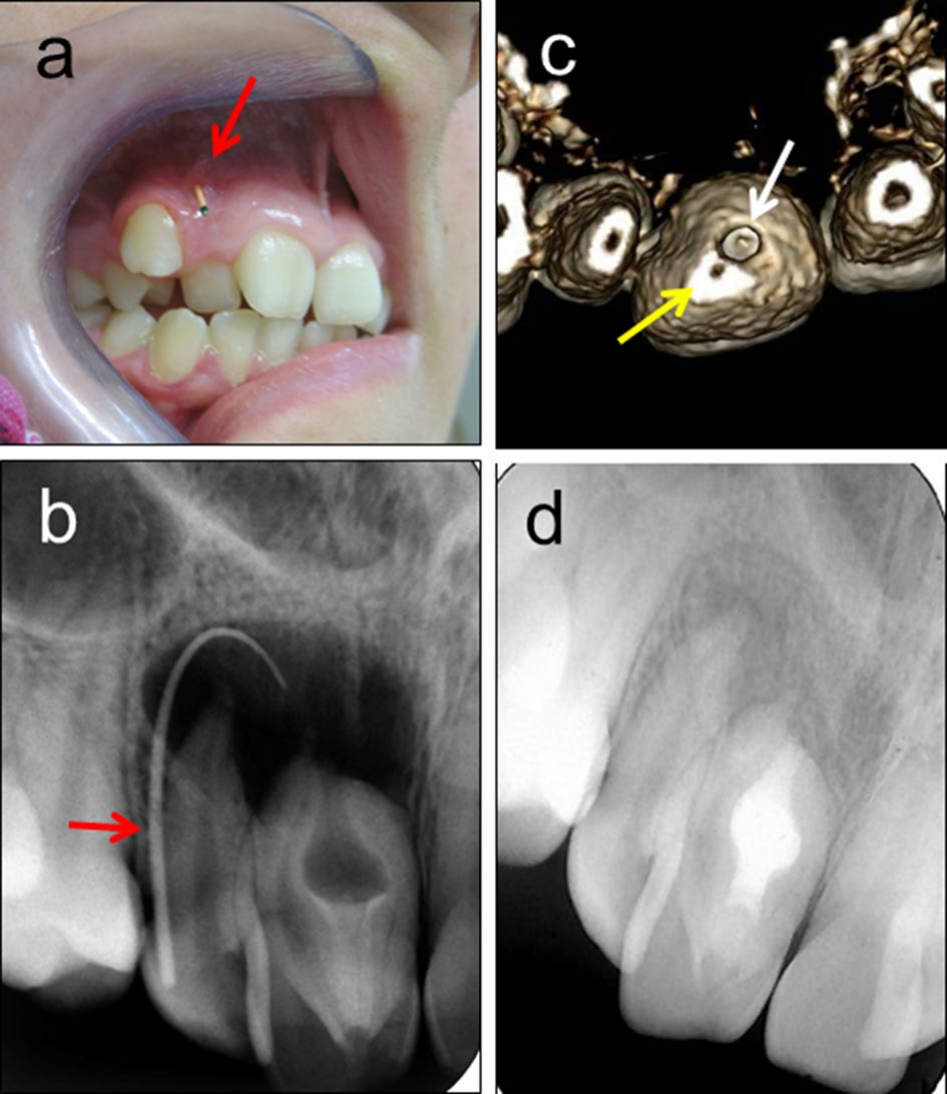
A rare case of Type III dens invaginatus with complications. This rare case of dens invaginatus was not diagnosed until the patient presented with a large periapical lesion with sinus tract [(**a**,**b**) Red arrows: a gutta percha point inserted into a sinus tract]. Both teeth #11 and #12 responded positively to cold stimuli, indicating vital pulps. The lesion resulted from bacterial contamination of the lumen of the Type III dens invaginatus which had an independent apical opening [(**c**) 3D reconstruction from CBCT]. Yellow arrow: the apical opening of the root canal. White arrow: apical opening of the dens invaginatus. Disinfection and obturation of the lumen of the dens invaginatus allowed for healing of the periapical lesion (**d**). Both teeth remained vital. (Adapted from: Kfir et al. *International Endodontic Journal*, 46:275–288, 2013).

Preventive measures consist mainly of sealing the entrance to the invagination to prevent bacterial penetration and proliferation in the “sanctuary” of the invagination^10^. Failure to diagnose a case of dens invaginatus and failure to apply the necessary preventive measures may often lead to involvement of the pulp resulting in pulp infection and necrosis and development of a periapical lesion (Fig. 2)^4,10^. When involvement of the pulp occurs, root canal treatment may be required to prevent involvement of the periapical bone or the need for surgical endodontic approach^4,10^ (Figs. 2 and 3).

Minor forms of Type I dens invaginatus may have limited depth and may be kept free of bacterial biofilm by conventional oral hygiene procedures. Nevertheless, unless a radiograph is taken, one cannot be certain of the depth of a specific invagination^9,10^. In cases of deep invagination, the invagination may serve as a sanctuary for bacteria, and caries may develop in the invagination, unnoticed until clinical symptoms appear^10^.

Dens invaginatus may also present a local inherent weakness in the enamel, as the invaginated enamel is often hypo-mineralized, which may make it more susceptible to deterioration^10,12^. Furthermore, both enamel and dentin may be thinner in the invagination than in the crown, and the dentin may contain strains of vital connective tissue or even fine canals with communication with the dental pulp^13^. Consequently, early pulp involvement may occur, with pulp infection and necrosis developing within a few years of eruption, sometimes even before root-end closure^10,12,14,15^.

Cases with a very deep invagination are classified as Type II dens invaginatus^10^ (Fig. 1b,d) and may present a substantial endodontic challenge when pulp involvement occurs. Conventional endodontic therapy through or next to an enamel-lined passage or structure may be difficult.

The above indicates that early, simple preventive measures, such as sealing the entrance to the invagination as soon as it is detected, may prevent the above complications^16^.

Type III dens invaginatus was not observed in the present study, most likely because it is a rare phenomenon; nevertheless, this anomaly has been noted in sporadic case reports^7,17–19^. Cases of Type III dens invaginatus may result in rather complicated and challenging situations (Fig. 3), such as those reported by Kfir et al.^7^, Brooks and Ribera^17^, Fregnani et al.^18^ and Solomonov et al*.*^19^. It is most likely that the abnormal morphological features that were found in the present study may also be used for the early prediction and detection of such complicated and challenging situations.

Previously published data regarding the prevalence of dens invaginatus may be divided into two major groups: a group of studies in which dens invaginatus prevalence in the whole dentition is reported and a group in which dens invaginatus prevalence in maxillary incisors was studied and reported. When the whole dentition is studied, the prevalence of dens invaginatus is usually reported as a percentage of the patients who presented with dens invaginatus, which may range from 2% of the patients^15,20,21^ up to 10% and even 31% of the patients^20,22,23^. Such data are not comparable with the results of the present study.

Similar to the present study, the researchers in the second group chose to concentrate specifically on the maxillary incisors, because the permanent maxillary incisors appear to be the teeth most frequently affected by this anomaly^1^.

Among the studies focused on the maxillary incisors (Table 2), one can also find a range of prevalence from as low as 0.3–5%^24–27^ to as high as 32% or even 75%^2,3,28,29^. Some of these differences may be attributed to ethnic background, but comparison of the results of Miyoshi et al.^28^ (38%) with those of Fujiki et al.^27^. (4%) and Gotoh et al.^11^ (10%) indicate otherwise. The latter three studies were all conducted on Japanese population, which has a relatively uniform ethnic background, yet the findings in these studies are substantially different.

**Table 2:** Prevalence of dens invaginatus in maxillary incisors.

Authors (ref)	Year	Sample	Country	Method	Teeth	Frequency of DI
Atkinson^30^	1943	500 T	Mexico	PA-R	Maxill. lateral incisors	10% of teeth
Boyne^24^	1952	1000 T	USA	PA-R	Maxill. incisors	0.3% of teeth
Stephens^25^	1953	150 P 300 T	USA	PA-R	Maxill. lateral incisors	8% of patients 5% of Maxill. lateral incisors
Hallet^3^	1953	400 P 1600 T	USA	Clinical observation	Maxill. incisors	40% of patient
Grahnen et al.^26^	1959	3020 T	Sweden	PA-R	Right Maxill. incisors	3% of patients
Miyoshi et al.^28^	1971	1223 T	Japan	Extracted teeth—Radiographs ***	Maxill. lateral incisors	39% of teeth
Fujiki et al.^27^	1974	2126 T	Japan	PA-R	Maxill. lateral incisors	4% of teeth
Gotoh et al.^11^	1979	766 T	Japan	PA-R	Maxill. lateral incisors	10% of teeth
Shi et al.^29^	2013	67 P ** Ancient people	China	Micro-CT	Full-mouth surveys	31% of patients 59% of Maxill.lateral incisors
Capar et al.^2^	2015	300 P	Turkey	CBCT versus P-R	Full-mouth surveys	P-R: 3% of patient CBCT: 11% of patients 75% of Maxill. lateral incisors
Ceyhanli et al.^31^	2015	2067P	Turkey	CBCT	Full-mouth surveys	2.8% of teeth
Różyło et al.^5^	2017	33P	Poland	CBCT	Full-mouth surveys	53.7% of teeth
Kfir et al.	Present study	1621 T 523 P	Israel	PA-R + Clin.photographs	Maxill. incisors	24% of patients 26% of teeth

*T* teeth, *P* patients, *Maxill.* Maxillary, *PA-R* periapical radiographs, *P-R* Panoramic radiographs, *CBCT* Cone beam CT.

**Ancient people.

*** Extracted maxillary lateral incisors, which allowed an additional mesio-distal.

Another potential reason for the high variability in the reported prevalence of dens invaginatus in maxillary incisors could be the method and criteria used to define the presence of dens invaginatus in the incisors. Mild cases of type I dens invaginatus, such as those presented in Fig. 1e,f, may be overlooked when only periapical radiographs are evaluated, thus affecting the results.

It appears that studies in which methods other than simple periapical radiographs were used for diagnosis, report the most reliable information. When taking radiographs of extracted lateral incisors, Myoshi et al.^28^ could also take a mesio-distal projection, thereby identifying dens invaginatus in 39% of the maxillary lateral incisors in same Japanese population which was evaluated by Fujiki et al.^27^. and Gotoh et al.^11^, who reported a much lower prevalence (4% and 10%, respectively) for dens invaginatus, which was determined using periapical radiographs alone^11,27,28^. Shi et al*.* studied ancient man samples and used micro-CT, which likely provided more reliable 3D information of the lateral incisor structure; they reported an incidence of 59%^29^. Similarly, Capar et al*.* used cone-beam CT (CBCT) and identified dens invaginatus in 75% of the maxillary lateral incisors^2^. The same group used also panoramic radiographs in which they identified dens invaginatus in only 3% of the patients, whereas CBCT of the same patients led to the detection of dens invaginatus in 11% of the patients. Thus, CBCT is a more sensitive technique for the detection of dens invaginatus.

One limitation of the present study is the fact that the young Israeli population that was studied likely possessed a mixed ethnic background due to massive immigration during the last seven decades; thus, the results may not be comparable with population with a more uniform ethnic background. Another limitation is that periapical radiographs were used instead of CBCT in the present study. This should have been avoided since the presently available CBCT technology would subject the study cohort to unjustified additional radiation exposure. Furthermore, to conduct the present study high quality photographs of the crowns were required but the database available to the present study did not include CBCT scans, which were not conducted as a screening method in the Orthodontic Clinic in our School.

An interesting finding in the present study was the presence of many cases with unilateral dens invaginatus, which may support the theory that local factors are involved in the formation of this anomaly^30,32,33^.

The clinical morphological features that were evaluated in the present study (Table 1) were chosen from a list of features that have been previously sporadically reported to be associated with dens invaginatus^1,4,6^. However, our sample size was too small to identify statistically significant correlations between dens invaginatus and each of these individual anatomical features. Nevertheless, when evaluated together, as a group, these morphological features could predict the presence of dens invaginatus (*p* < 0.05).

Radiographs of the anterior teeth are not recommended for the routine screening of children and young adults, due to concerns regarding unnecessary exposure to radiation. However, the clinical implications of the present study suggest that a thorough clinical examination of maxillary incisors should be routinely performed. If a pediatric dentist observes one of the morphological features described here (Table 1), it may be his/her obligation and duty to take a radiograph of the affected teeth to verify the presence or absence of dens invaginatus and determine its extent, thereby allowing the application of preventive measures before any pathological changes occur.

## Conclusions

1. Dens Invaginatus is common in the study population in maxillary incisors, especially lateral incisors.
2. A group of clinical morphological features may predict the presence of dens invaginatus.
